# Revealing the Antiperspirant Components of Floating Wheat and Their Mechanisms of Action through Metabolomics and Network Pharmacology

**DOI:** 10.3390/molecules29030553

**Published:** 2024-01-23

**Authors:** Shengnan Dong, Qing Tian, Ming Hui, Shouyu Zhang

**Affiliations:** 1 College of Biological Engineering, Henan University of Technology, Zhengzhou 450001, China; dongshengnan@stu.haut.edu.cn; 2 Industrial Microorganism Preservation and Breeding Henan Engineering Laboratory, Zhengzhou 450001, China; tianqing@haut.edu.cn; 3 College of Smart Health, Henan Polytechnic, Zhengzhou 450046, China

**Keywords:** floating wheat, hyperhidrosis, plant metabolomics, network pharmacology, molecular docking

## Abstract

Floating wheat is a classical herbal with potential efficacy in the treatment of hyperhidrosis. Aiming at revealing the main components and potential mechanisms of floating wheat, a comprehensive and unique phytopharmacology profile study was carried out. First, common wheat was used as a control to look for chemical markers of floating wheat. In the screening analysis, a total of 180 shared compounds were characterized in common wheat and floating wheat, respectively. The results showed that floating wheat and common wheat contain similar types of compounds. In addition, in non-targeted metabolomic analysis, when taking the contents of the constituents into account, it was found that there indeed existed quite a difference between floating wheat and common wheat and 17 potential biomarkers for floating wheat. Meanwhile, a total of seven components targeted for hyperhidrosis were screened out based on network pharmacology. Seven key differential components were screened, among which kaempferol, asiatic acid, sclareol, enoxolone, and secoisolariciresinol had higher degree values than the others. The analysis of interacting genes revealed three key genes, namely, MAP2K1, ESR1, and ESR2. The Kyoto Encyclopaedia of Genes and Genomes (KEGG) and Gene Ontology (GO) enrichment analyses showed that various signaling pathways were involved. Prolactin signaling, thyroid cancer, endocrine resistance, gonadotropin secretion, and estrogen signaling pathways were the main pathways of the intervention of floating wheat in excessive sweating, which was associated with the estrogenic response, hormone receptor binding, androgen metabolism, apoptosis, cancer, and many other biological processes. Molecular docking showed that the screened key components could form good bindings with the target proteins through intermolecular forces. This study reveals the active ingredients and potential molecular mechanism of floating wheat in the treatment of hyperhidrosis and provides a reference for subsequent basic research.

## 1. Introduction

Hyperhidrosis is a typical symptom of menopausal syndrome, which is a neurological dysfunction caused by the decline in hormone levels during menopause accompanied by troublesome and progressive physical and psychological symptoms. Clinical treatment of hyperhidrosis is difficult due to the high frequency of the disease, which seriously affects the quality of life. Therefore, a treatment for hyperhidrosis needs to be developed [1,2]. Floating wheat is an immature wheat grain that appears shriveled because of early harvesting and drying processing. A shriveled wheat kernel is an inexpensive and readily available herbal medicine, and several studies have shown that floating wheat plays an important role in the treatment of hyperhidrosis [3,4,5,6]. It also has a positive effect on other complications caused by hyperhidrosis [7,8,9]. However, the specific mechanism by which floating wheat alleviates hyperhidrosis is unclear. Further studies are required to identify the specific disease targets of the components of floating wheat.

Comprehensive and systematic characterization of metabolites in floating wheat is a difficult task due to trace concentrations in vivo, the unpredictability of metabolites, the interference of endogenous substances, and a lack of standards. Conventional methods of manually and consciously searching for differences between control and treatment chromatograms may artificially lose some components, especially some metabolites, in vivo [10]. Plant metabolomics is an efficient, multi-targeted technique for studying various small-molecule metabolites in plants and their dynamic changes at a given stage, which offers access to comprehensively characterizing the chemical components among different species and parts of plants. In recent years, plant metabolomics based on liquid chromatography-mass spectrometry has attracted much attention in the field of plants and has been widely applied to the study of traditional Chinese medicine (TCM) [11,12,13,14,15].

Network pharmacology is a new discipline that combines system biology and system pharmacology to study disease and drug mechanisms of action. The aim is to explain the mechanism of drug action in a multilevel, systematic way, similar to the treatment concept of TCM dialectic therapy. Given the complexity of TCM components, multiple targets of action, and wide therapeutic signaling pathways, an in-depth study of TCM treatment is challenging. The use of network pharmacology technology provides a basic and scientific explanation for the study of TCM problems [16,17,18,19,20].

**Figure 1:**
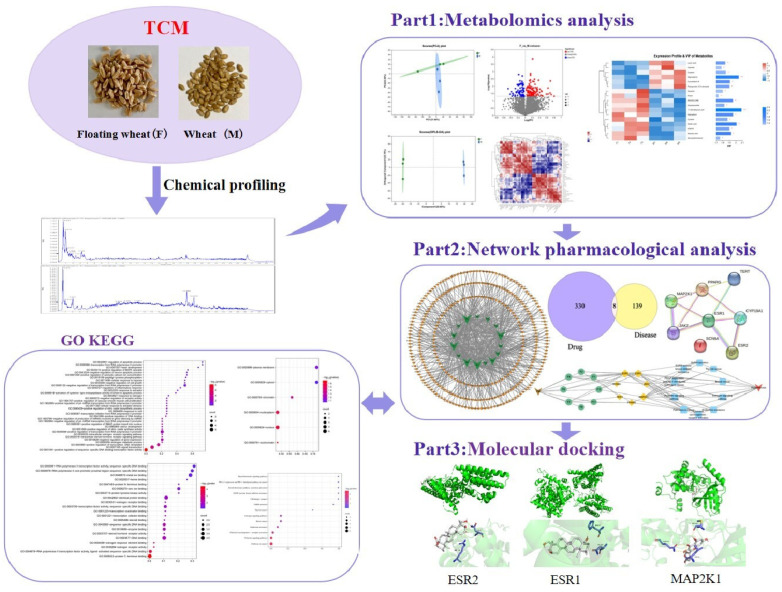
General flow chart.

In this study, we screened and analyzed differential metabolites of floating wheat and wheat by plant metabolomics and analyzed the major differential metabolites in combination with targets of hyperhidrosis using network pharmacology. Component–target networks and protein–protein interaction (PPI) networks of component–disease interaction targets were constructed. Also, in order to investigate the signaling pathways for their biological functions and efficacy, Gene Ontology (GO) and Kyoto Encyclopedia of Genomes (KEGG) enrichment analyses were performed. Then, the functional components and main targets were validated by molecular docking techniques. This study revealed the potential mechanism through which floating wheat inhibits the development of hyperhidrosis during menopause, and the detailed workflow is shown in Figure 1.

## 2. Results and Discussion

### 2.1. Metabolomic Analysis

**Figure 2:**
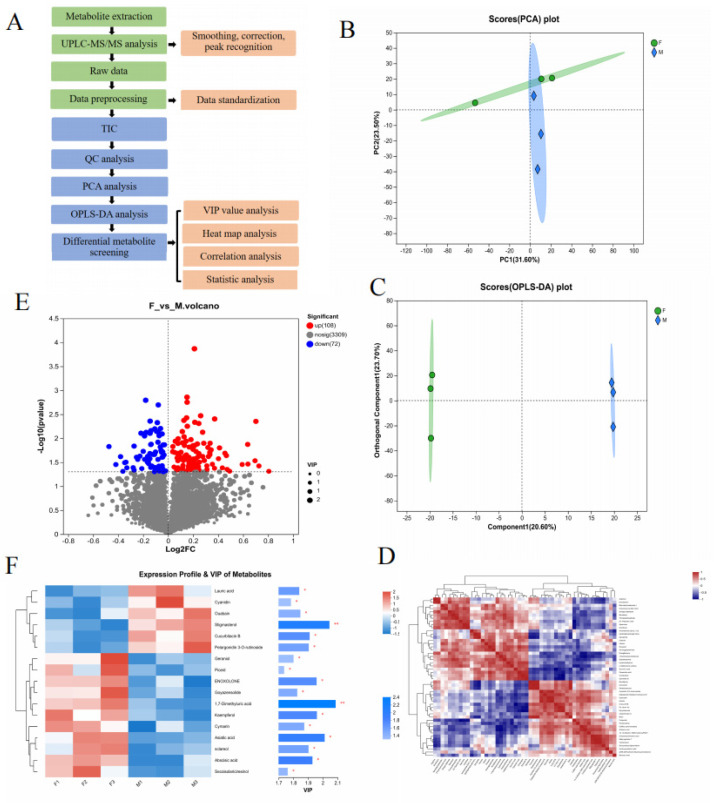
Differences in metabolites between Groups F and M. (**A**) Workflow of metabolomic analysis. (**B**,**C**) The PCA and OPLS-DA models can effectively distinguish differences between groups. (**D**) Correlation analysis of different components. (**E**) A total of 108 metabolite ions have upregulated expression and 72 metabolite ions have downregulated expression in the volcanic map. (**F**) The VIP value of secondary metabolites of different plants is >1 (* *p* < 0.05, ** *p* < 0.01).

A metabolomic analysis was performed on floating wheat (F) and wheat (M) samples by ultrahigh-performance liquid chromatography–tandem mass spectrometry (UPLC-MS/MS) in net and pos modes, respectively, and the metabolites in F and M were separated within 40 min (Figure 2A exhibited the workflow). Ionic characterization was detected by the peak-to-peak ratio, and 180 metabolites were screened (details are provided in the attached table), which showed that 102 ions were upregulated and 78 ions were downregulated and identified based on the retention time (the correlation heat map and volcano plot are shown in Figure 2D,E). In the advanced data analysis, cluster analysis (heat map) (Figure 2F) vividly showed differential metabolites in floating wheat. The MS and MS/MS spectra were compared with metabolomic databases and references, including alkaloids and their derivatives, amino acids, organic acids, terpenoids (e.g., carotenoids and steroids), and compounds (e.g., flavonoids and phenolic acids). The result shows similarity with the chemical species identified in durum wheat by Hédia M. et al. [21].

Floating wheat (F) and wheat (M) were different types of wheat. They had great similarities in appearance and chemical composition, but the contents of these chemicals differed. To date, few studies have used chemical markers to distinguish between the two components. The QC data were analyzed using PCA to show the trend of metabolome separation between the two groups and the variability of metabolomes present between the sample groups [22]. The PCA analysis is given in Figure 2B. In the PCA analysis, the first two principal components explained 55.1% of the total variance (31.6% and 23.5%). The F samples could be separated from the M ones, indicating that the PCA method is effective for the identification of F samples.

To find out the key markers that contribute most to the difference between GP and JP, an OPLS-DA analysis was performed. OPLS-DA allows access to maximal difference markers between metabolite groups [23]. Potential structural discriminant analysis (OPLS-DA) modeling of the variables was performed using regression coefficient plots with 95% confidence intervals, and metabolites with VIP values greater than 1 were selected as metabolite cutoffs. The results are shown in Figure 2C. A Student’s *t*-test was performed to examine whether the differences in metabolite concentrations were significant. A total of 17 key plant secondary metabolites with significant differences were screened in both samples (Table 1), of which 11 differential components were screened in the positive ion mode and 6 differential components were screened in the negative ion mode. The screened plant secondary metabolites were mainly flavonoids, terpenoids, and sterols, and the related VIP plots are shown in Figure 2F. Research has revealed that plant-derived compounds, such as flavonoids, terpenoids, and steroids, are structurally similar to endogenous estrogens in humans and can bind to estrogen receptors in the body, which are known as phytoestrogens (PEs) that have estrogen-like effects and exert a positive effect on the treatment of menopausal syndromes [24]. Based on the results, it appears that the flavonoids, terpenoids, and steroidal metabolite content of floating wheat is generally higher than that of wheat, which may be the key to the antiperspirant effect of floating wheat. Based on this, we next further analyzed the potential mechanism of the antiperspirant effect of floating wheat through the network pharmacology technique.

### 2.2. Component–Target Network Analysis

**Figure 3:**
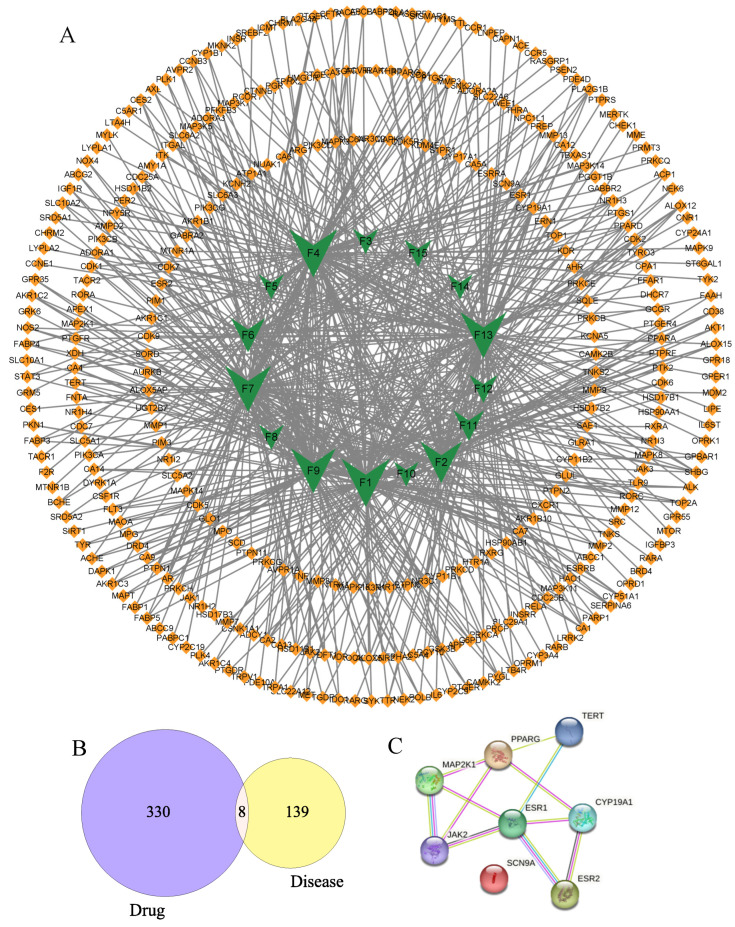
Component–target and PPI network diagrams.

Nowadays, so as to explore the multiple components, multiple targets, and various pathways of floating wheat against hyperhidrosis, network pharmacology is performed. First and foremost, in Swiss Target Prediction, parameters with probability values greater than 0.1 were set, and 338 targets were obtained for the remaining 15 components, excluding Geraniol and Goyazensolide, for which no relevant targets were found. Meanwhile, 147 hyperhidrosis targets with GDA scores greater than 0.1 were obtained from DisGeNET. Based on these data, a component–target network was constructed (Figure 3A). As shown in Figure 3A, the network consisted of 351 nodes and 634 interactions. Among them, 15 component-related targets had 8 overlapping targets with the hyperhidrosis targets (Figure 3B), and the targets, which were called potential hub proteins, were SCN9A, PPARG, MAP2K1, TERT, JAK2, CYP19A1, ESR1, and ESR2.

**Figure 4:**
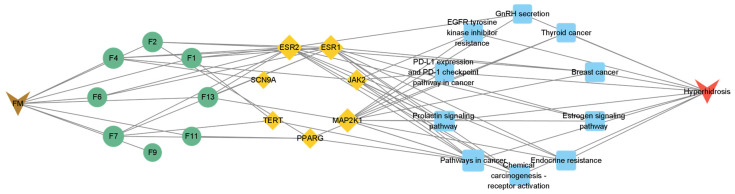
TCM–Metabolite–Gene Target–Metabolic Pathway–Disease Network Map.

**Figure 5:**
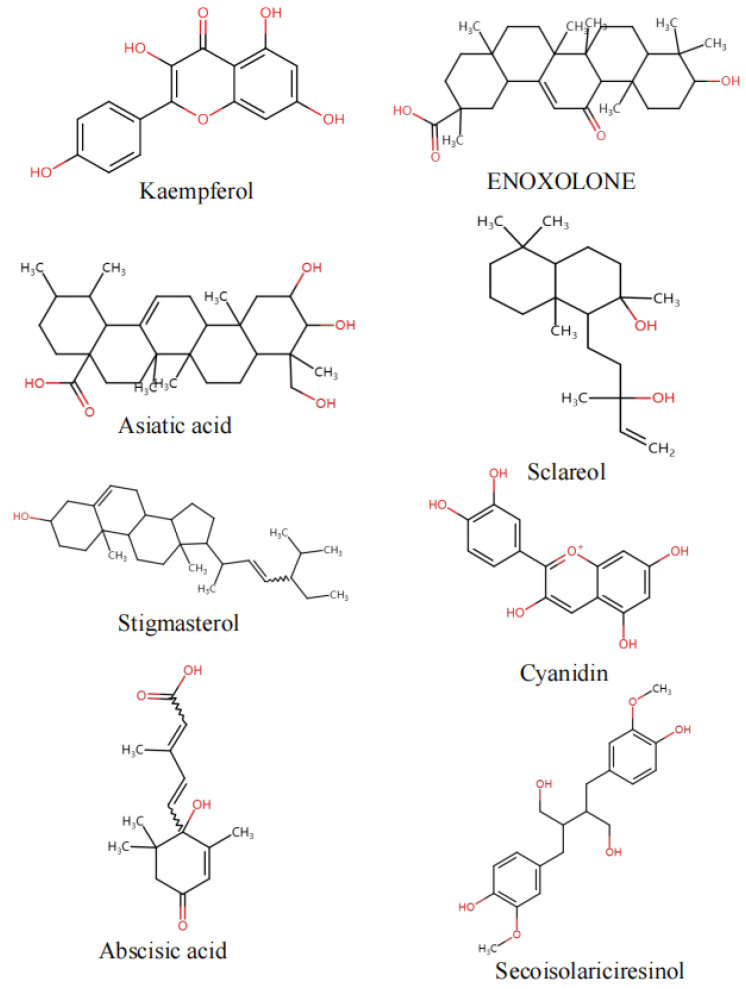
Key component structure chart.

To make a profound study of the mechanisms of floating wheat against hyperhidrosis, a network incorporating TCM, metabolites, metabolite-related genes, metabolic pathways, and diseases was constructed (Figure 4). Further analysis showed that in addition to cymarin, geraniol, 1,7-dimethyluric acid, ouabain, pelargonidin 3-O-rutinoside, piceid, cucurbitacin B, lauric acid, and goyazensolide, eight other compounds could target hyperhidrosis-related targets (Figure 5). Among them, the only cyanidin (F9)-related gene target is CYP19A1, which cannot be linked to hyperhidrosis-related metabolic pathways. A PPI network was established for the gene targets (Figure 3C), with results showing that JAK2, MAP2K1, ESR1, and ESR2 were the main targets. ESR1 and ESR2 are estrogen receptors. ESR1 has a well-defined role in maintaining life, regulating sexual function, and contributing to organismal development, immunomodulation, the treatment of skin disorders, and fertility control [25]. ESR2 can bind estrogen with a similar affinity to ESR1 and activate the expression of estrogen response element-containing reporter genes in an estrogen-dependent manner [26]. JAK2 (tyrosine-protein kinase) plays a key role in signal transduction in the cytoplasm through its association with the growth hormone, prolactin, leptin, erythropoietin, and thrombopoietin [27]. MAP2K1 (dual specificity protein kinase) is an important component of the MAP kinase signaling pathway.

**Figure 6:**
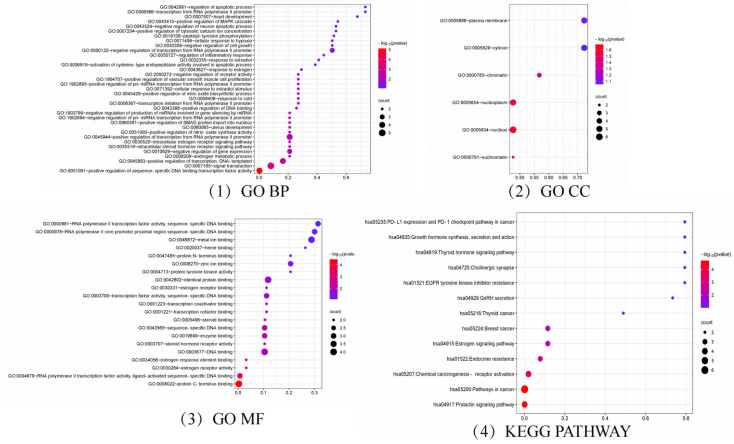
GO and KEGG analysis results.

To further clarify the relationship between targets and pathways, we analyzed target–pathway interactions by using data extracted from the DAVID database and screened for important pathways via KEGG analysis, with h-corrected *p*-values less than 0.05. The results showed that cancer, prolactin, and chemical carcinogenesis (receptor activation) signaling pathways might be the main pathways of the components acting on hyperhidrosis (Figure 6). Thus, floating wheat may inhibit hyperhidrosis through JAK2, MAP2K1, ESR1, and ESR2, affecting metabolite-related genes, which in turn indirectly affect signaling pathways such as cancer, prolactin, and chemical carcinogenesis (receptor activation), thereby inhibiting hyperhidrosis.

### 2.3. Analysis of GO and KEGG Results

GO and KEGG enrichment analysis of targets related to hyperhidrosis intervention by floating wheat using bioinformatics and visualization of the results (Figure 6). The GO enrichment analysis by BP, MF, and CC levels resulted in a total of 65 entries, with the *p*-values corrected using the Benjamini–Hochberg procedure in accordance with *p* < 0.05.

In the category of BPs, the target proteins were mainly involved in prostate gland growth, female genitalia development, the intracellular estrogen receptor signaling pathway, and the cellular response to estradiol stimulation. The MF class target proteins were mainly involved in estrogen response element binding, estrogen receptor activity, steroid hormone receptor activity, and estrogen receptor binding. In the CC class, the target proteins were categorized as chromatin and plasma membrane, nucleoplasm, cytoplasmic lysate, etc.

A total of 14 KEGG-enriched items with h-corrected *p*-values < 0.05, including the prolactin signaling pathway, endocrine resistance, GnRH secretion, estrogen signaling pathway, growth hormone synthesis, secretion, and action, were identified via KEGG analysis.

### 2.4. Molecular Docking Study

**Figure 7:**
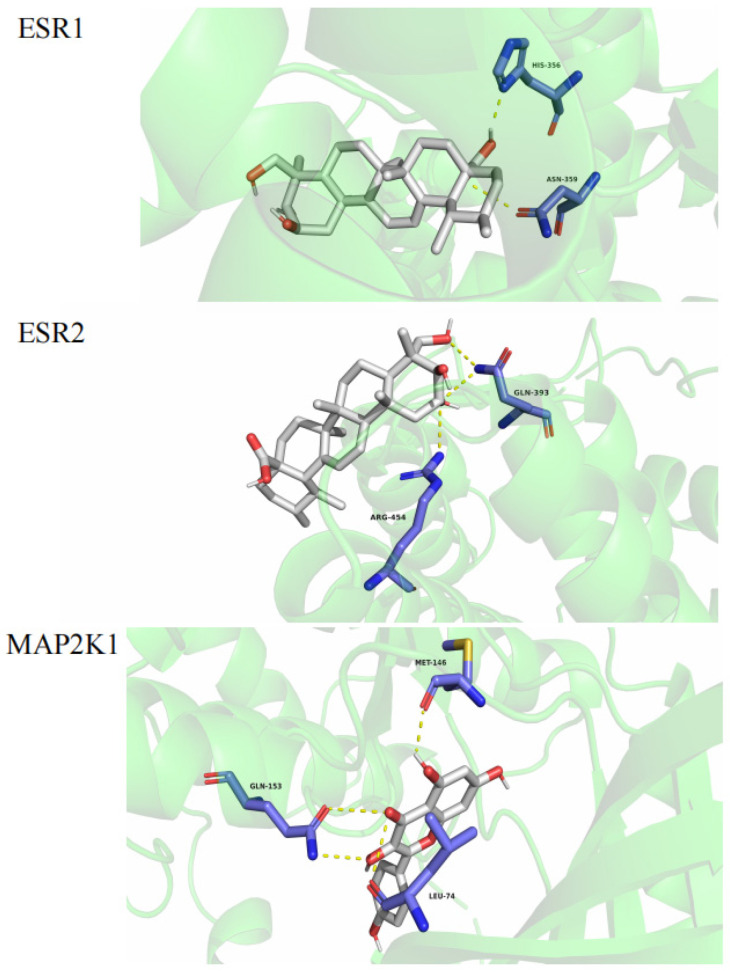
Molecular docking.

On the basis of network pharmacology, eight core compounds (mainly terpenoids and flavonoids) were screened as sclareol, asiatic acid, enoxolone, abscisic acid, secoisolariciresinol, kaempferol, stigmasterol, and cyanidin. MAP2K1, ESR1, and ESR2 were nodes in the interaction network, suggesting that they played key roles in the mechanism of action. The molecular docking of kaempferol with the key protein MAP2K1 and the molecular docking of abscisic acid with the key proteins ESR1 and ESR2 were verified. The docking scores of the compounds and proteins are shown in Table 2. Figure 7 presents the best docking image of the receptor and ligand after visualization.

The results indicated that kaempferol could interact with MAP2K1 (PDB: 5HZE). Asiatic acid could interact with ESR1 (PDB: 1A52) and ESR2 (PDB: 1L2J) to form three hydrogen bonds with MET-146, GLN-153, and LEU-74 in MAP2K1 and with ESR1 with HIS356 and ASN359 to form two hydrogen bonds. It could also interact with ESR2 via GLN393 and ARG454, as shown in Figure 7.

The lower the binding energy is, the higher the affinity between the receptor and ligand is, and the more stable the conformation is. Binding energy that is less than −5 kcal/mol indicates good ligand–receptor binding activity [24,28,29,30]. The molecular docking results showed that all the binding energies were less than –5 kcal/mol, indicating that the compounds had a high affinity for proteins. Kaempferol had the highest docking binding energy with MAP2K1 (−13.2 kcal/mol), and abscisic acid had the lowest docking binding energy with ESR2 (−7.3 kcal/mol). The compounds bound well to the three core targets (MAP2K1, ESR1, and ESR2), so we predict that all of the compounds may play a key role in the treatment of hyperhidrosis.

## 3. Materials and Methods

### 3.1. Materials and Laboratory Equipment

The natural dried plant materials used in the study (floating wheat and wheat) were obtained from Shangyuan Tang Pharmacy (Zhengzhou, China); CH3CN, CH3OH, HCOOH, and CH3COONH4 were obtained from McLean Company (Shanghai, China), and ultrapure water was prepared by a laboratory water maker, the manufacturer of which was China Colton Water Co. (Beijing, China). In addition, an UltiMate 3000 Ultra High-Performance Liquid Chromatograph (UHPLC), Thermo Fisher Scientific, (Waltham, MA, USA), an X500R QTOF High-Resolution Mass Spectrometer (HRMS), AB SCIEX, (Boston, MA, USA), and an ACQUITY UOLC HSS T3 Chromatographic Column (Voltas), Waters, (Milford, MA, USA), were also used.

### 3.2. Sample Pretreatment

The samples were mixed well, placed in a mortar, added to liquid nitrogen, and ground into powder. Exactly 0.1 g of the powder was weighed, placed in a 1.5 mL microcentrifuge tube, added with 600 μL of 75% methanol, subjected to vortex mixing for 60 s, placed in ultrasonic iced water for 30 min, and centrifuged at 4 °C and 17,000 r/min for 15 min. The supernatant was taken and put in a 1.5 mL microcentrifuge tube at 35 °C vacuum centrifugation to dry. Then, 100 μL of 50% methanol was added to water, vortexed for 60 s to fully dissolve the sample extract at 4 °C, and centrifuged at 17,000 r/min for 15 min. The sample extract was obtained by centrifugation at 4 °C and 17,000 r/min for 15 min.

### 3.3. UPLC-MS/MS

Chromatographic conditions: An UltiMate 3000 ultrahigh-performance liquid chromatograph was used with an ACQUITY UOLC HSS T3 column (1.8 µm, 2.1 mm × 100 mm) with 0.1% formic acid aqueous solution (A) and 0.1% formic acid acetonitrile (B) as the mobile phases in the positive ionization mode and water (2 mM ammonium acetate, A)-acetonitrile (B) in the negative ionization mode. In addition, 0.1% (2.1 mm × 100 mm, 1.7 μm) formic acid aqueous solution (A)-acetonitrile (B) was the mobile phase, and gradient elution was performed (0–1.5 min, 95% A, 5% B; 1.5–2.5 min, 95% A, 5% B; 2.5–14 min, 90–60% A, 10–40% B; 14–22 min, 60–5% A, 40–95% B; 22–25 min, 5–5% A, 95–95% B; 25–26 min, 5–95% A, 95-5% B; and 26–30 min, 95–95% A, 5–5% B). The column temperature was 40 °C, the injection volume was 5 μL, and the flow rate was 0.4 mL min^−1^.

Mass spectrometry conditions: The AB X500R Triple TOF mass spectrometer was used to perform primary and secondary mass spectrometry data acquisition on the basis of the data-dependent acquisition (IDA) function. In each data acquisition cycle, the strongest molecular ions with an intensity greater than 100 Da were selected to collect the corresponding secondary mass spectral data. The primary acquisition range was 50–1200 Da, the bombardment energy was 30 eV, and 10 secondary spectra were obtained every 50 ms. The electrospray ionization (ESI) ion source parameters were set as follows: atomization air pressure (GS1): 60 Psi, auxiliary air pressure: 60 Psi, air curtain air pressure: 35 Psi, temperature: 650 °C, and spray voltage: 5000 V in the positive ion mode and −4000 V in the negative ion mode.

Data analysis: The data were converted to .abf format by using AnalysisBaseFileConverter 2.74, and data standardization was outperformed on the converted .abf file by using MSDIAL version 4.6 software. Metlin, MassBank, MoNA, and HMDB databases were independently integrated based on first- and second-level mapping searches with version V6.0 to obtain the identification results. The basic data were analyzed by total ion chromatography. Principal component analysis (PCA) was used for quality control analysis, and orthogonal partial least squares discriminant analysis (OPLS-DA) was employed to observe the classification among different groups. Ions with a variable importance projection (VIP) > 1 and a *p*-value < 0.05 were considered differentiated metabolite ions. The HMDB database and related works of literature were searched with accurate quality to identify the differential metabolites. The compounds were identified within 5 ppm mass accuracy.

### 3.4. Component–Target Network Construction

Network analysis component targets were retrieved from Swiss Target Prediction (http://www.swisstargetprediction.ch/, accessed on 25 July 2023), an online target prediction platform [31]. Disease-related genes were screened by DisGeNET (https://www.disgenet.org/, accessed on 25 July 2023) [32]. PPIs were explored using the STRING database (version 11.5, https://string-db.org/, accessed on 25 July 2023), and protein interactions with confidence scores >0.4 were selected in the design settings after eliminating duplicates [33]. The chemical target and PPI networks were constructed using Cytoscape software (version 3.9.0) [34]. The above network analysis was conducted on 15–25 July 2023.

### 3.5. GO and KEGG Pathway Analyses

GO and KEGG data were collected using the DAVID (https://david.ncifcrf.gov/, accessed on 25 July 2023) database to clarify the role of the target proteins’ interaction with floating wheat target genes in gene function and signaling pathways [35]. GO analysis is used to screen biological processes (BPs), cellular components (CCs), and molecular functions (MFs) [36], and KEGG enrichment analysis can identify important signaling pathways involved in BPs. Subsequently, GO and KEGG data were uploaded to the Bioinformatics (http://www.bioinformatics.com.cn/, accessed on 25 July 2023) platform for visualization and analysis.

### 3.6. Molecular Docking

Molecular docking is a drug design technique for studying receptor and ligand interactions. It uses recognition and a theoretical simulation method of examining intermolecular interactions. It also predicts their binding modes and affinities [37]. It is used to observe whether the core components of floating wheat have been identified through metabolomics screening and network pharmacology and if they have been bonded to the core proteins or not.

On the basis of the results of the preliminary web-based pharmacological screening, the 3D structure of the target protein was retrieved from the Protein Data Bank (PDB) (http://wwwrcsb.org/, accessed on 25 July 2023) [38]. The PDB format file was downloaded. The protein was dehydrogenated and hydrogenated in AutoDock4 [39] and subsequently designated as a receptor; the structure was saved as a PDBQT protein receptor file. The drug molecular structures were downloaded from the PubChem (https://pubchem.ncbi.nlm.nih.gov/, accessed on 25 July 2023) database [40]. Similarly, in AutoDock4, we dehydrogenated the molecules, hydrogenated them, set the drug as a ligand, automatically configured the torsion tree in the software, and exported it as a ligand file in PDBQT format. The receptor and ligand PDBQT structures were then imported into AutoDock4 to define the molecular docking range. With the target protein as the grid center, the center coordinates (center x/y/z) and box size (size x/y/z) parameters were adjusted to ensure that the protein was completely covered by the grid box [20]. Molecular docking was accomplished by detecting the protein macromolecules, inserting tiny drug molecules, and configuring the manipulation method and docking parameters in AutoDock4. The PDBQT format was used to calculate the minimum binding energy. OpenBabel 3.1.1 software [41] was employed to convert the combined PDBQT format to the PDB format. Then, the docked complexes of the selected components were visualized using PyMoL 2.4 software [42].

## 4. Conclusions

In this study, the main bioactive metabolites and potential mechanisms of floating wheat for the treatment of menopausal hyperhidrosis were previously explored based on plant metabolomics and network pharmacology combined with molecular docking technology. The results of plant metabolomics analysis showed that there are 17 main potential active components of floating wheat, namely: kaempferol, asiatic acid, cyclamarin, sclareol, 1,7-dimethyluric acid, stigmasterol, enoxolone, oubain, cyclanidin, pelargonidin 3-O-rutinoside, abscisic acid, piceid, secoisolariciresinol, cucurbitacin B, lauric acid, geraniol, and goyazensolide. The mechanistic correlation of specific components and hyperhidrosis targets of floating wheat through network pharmacology reveals that vanillol, cumaric acid, ENOXOLONE, abscisic acid, secoisolariciresinol, kaempferol, and stigmasterol have a modulatory effect on hyperhidrosis, and protein targets ESR1, ESR2, and MAP2K1 were found to be the potential targets of floating wheat for the treatment of hyperhidrosis in menopause. Protein targets, ESR1 and MAP2K1, act through prolactin, thyroid cancer, endocrine resistance, GnRH secretion, and estrogen signaling pathways. 

Hyperhidrosis is most commonly seen in women who have an imbalance of hormone levels in their bodies during menopause, a condition caused by lower levels of estrogen. Among the specific components screened, secoisolariciresinol and kaempferol have estrogen-like effects; stigmasterol is mostly used for steroid hormone synthesis; and sclareol, asiatic acid, enoxolone, and abscisic acid also have a modulating effect on the body. Plant-based metabolomics and network pharmacology studies suggest that floating wheat may affect three candidate targets (ESR1, ESR2, and MAP2K1) via seven differential metabolites, thereby affecting disease-associated protein targets and 14 metabolic pathways. Meanwhile, we performed molecular docking to verify the intermolecular interactions and predict the binding modes and affinities, and the results demonstrated favorable molecular docking results. This study serves as a reference for further exploration of the mechanism of action of floating wheat in the treatment of menopausal hyperhidrosis and provides a basis for the development of drugs for the treatment of menopausal hyperhidrosis. However, further in vitro and in vivo studies are needed to investigate the mechanism of action of floating wheat and its bioactive components in the treatment of hyperhidrosis. The current research on the treatment of hyperhidrosis by floating wheat and its bioactive ingredients is still in the preliminary stage.

## Figures and Tables

**Table 1:** Differences in composition between wheat and floating wheat.

No.	Identification	tR (min)	Ion Mode	Formula	Measured *m*/*z*	Theoretical *m*/*z*	MS Fragments	VIP Value	*p* Value	Structure Types	Source
F1	Kaempferol	8.887	[M + H]+	C15H10O6	287.05579	287.05502	61.0394, 69.0701, 111.1251, 269.2288, 287.0516, 287.0568	1.958	0.01738	Flavonoids	F, M
F2	Asiatic acid	15.804	[M + Na]+	C30H48O5	511.32941	511.33939	300.1110, 349.2677, 449.3287, 511.2344, 511.2828, 511.3082, 511.3290, 511.3867	2.0109	0.01147	Triterpenoids	F, M
F3	Cymarin	14.853	[M + H]+	C30H44O9	549.30524	549.30579	550.3086, 551.3120	1.8719	0.03239	Others	F, M
F4	Sclareol	13.925	[M + H − H2O]+	C20H36O2	291.27084	291.2688	55.0529, 57.0687, 69.0690, 79.0565, 81.0701, 93.0666, 93.0725, 95.0840, 98.5628, 107.0848, 109.1009, 118.0788, 123.0839, 132.0581, 133.1004, 135.1157, 141.0690, 149.1010, 161.0966, 175.1505, 195.1757, 245.1874, 250.0731, 263.1998, 273.1864, 291.1551, 291.1969, 291.2718	1.9011	0.0278	Diterpenoid	F, M
F5	1,7-Dimethyluric acid	8.317	[M + H]+	C7H8N4O3	197.07199	197.06691	53.0353, 65.0410, 69.0698, 77.0393, 85.0622, 91.0511, 93.0694, 95.0471, 95.0819, 105.0685, 107.0836, 109.0987, 119.0835, 123.1155,128.0610, 131.0839, 133.1003, 135.1167, 137.0957, 140.0525, 151.1129, 155.1074, 161.0964, 171.0120, 179.1072, 197.0722, 197.1181	2.0852	0.004227	Steroids	F, M
F6	Stigmasterol	21.832	[M + H]+	C29H48O	395.36157	395.36722	396.3649, 397.3682	2.0434	0.007862	Steroids	F, M
F7	Enoxolone	19.489	[M + H]+	C30H46O4	471.34607	471.34689	325.0317, 469.7909, 471.0971, 471.3142	1.9558	0.01902	Triterpenoids	F, M
F8	Ouabain	8.012	[M + H]+	C29H60O20	585.29474	585.29102	585.2968	1.8457	0.03718	Steroids	F, M
F9	Cyanidin	7.823	[M]+	C15H11O6	287.04913	287.05447	66.9806, 69.0682, 89.0590, 105.0666, 111.1155, 202.0814, 283.0601, 287.0614	1.7815	0.04971	Others	F, M
F10	Pelargonidin 3-O-rutinoside	6.722	[M]+	[C27H31O14]+	579.17236	579.17078	549.1657, 561.1414, 579.1728	1.9023	0.02756	Others	F, M
F11	Abscisic acid	9.078	[M − H]−	C15H20O4	263.1319	263.1283	136.0574, 137.0663, 143.0734, 148.0572, 153.091, 161.1010, 163.0785, 189.1001, 201.1309, 204.1161	1.9278	0.01339	Organic acid	F, M
F12	Piceid	3.831	[M − H]−	C20H22O8	389.12888	389.12271	59.0125, 65.3812, 85.0703, 89.0241, 101.0230, 117.0439, 121.0454, 123.0454, 128.0361, 134.0366, 135.0439, 138.0333, 149.0596, 150.0318, 151.0397, 153.0543, 154.0292, 158.0407, 165.0587, 171.0766, 177.0163, 178.0279, 181.0443, 191.0726, 193.0511, 209.1208, 227.1399, 267.0635, 279.0677, 282.0882, 294.0857, 297.1192, 303.1496, 312.0914, 312.1022, 327.1270, 345.1371, 389.1862	1.7359	0.04879	Polyphenols	F, M
F13	Secoisolariciresinol	8.654	[M − H]−	C20H26O6	407.17181	407.17114	57.0342, 73.0289, 80.9415, 85.0344, 89.0221, 127.9367, 145.0341, 145.0685, 149.1069, 161.0283, 163.0796, 165.0953, 175.1069, 188.9399, 191.0908, 191.0922, 194.2182, 197.0443, 209.0041, 227.1967, 234.0806, 245.1406, 245.1470, 253.0777, 267.0733, 269.1503, 271.2068, 283.1877, 284.0556, 287.1893, 289.0115, 303.2969, 321.1612, 371.2426, 396.0325, 407.0913, 407.1902, 407.2478, 407.2561, 407.2623, 407.2643, 407.2705, 407.2767, 407.2993	1.7599	0.04208	Terpenoids	F, M
F14	Cucurbitacin B	11.48	[M − H]−	C32H46O8	557.30316	557.31201	558.3065, 559.3098	1.909	0.01928	Terpenoids	F, M
F15	Lauric acid	20.208	[M − H]−	C12H24O2	199.17165	199.17062	68.9935, 162.8397, 169.9996, 181.9982, 199.1714	1.8381	0.02488	Others	F, M
F16	Geraniol	17.201	[M + H − H2O]+	C10H18O	137.13045	137.133	55.0128, 55.0522, 57.0310, 67.0575, 69.0691, 79.0547, 81.0316, 81.0435, 81.0711, 91.0531, 93.0696, 93.0745, 94.06378, 95.0572, 95.0632, 95.0891, 107.0753, 108.5005, 137.0217, 137.0982, 137.1090	1.7995	0.04855	Monoterpenes	F, M
F17	Goyazensolide	6.149	[M − H]−	C19H20O7	359.1062	359.11362	360.1095, 361.1129	1.8243	0.03397	Terpenoids	F, M

**Table 2:** Molecular docking results between the core components and key target proteins.

Target	Target (PDB ID)	Structure	Affinity (kcal/mol)
MAP2K1	5HZE	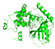	−13.2
ESR1	1A52	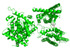	−8.5
ESR2	1L2J	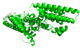	−7.3

## Data Availability

Data are contained within the article.
